# The pneumococcal response to oxidative stress includes a role for Rgg

**DOI:** 10.1099/mic.0.028282-0

**Published:** 2009-12

**Authors:** Magda E. Bortoni, Vanessa S. Terra, Jason Hinds, Peter W. Andrew, Hasan Yesilkaya

**Affiliations:** 1 Department of Infection, Immunity and Inflammation, University of Leicester, Leicester LE1 9HN, UK; 2 Department of Basic Sciences, Universidad De Monterrey, Monterrey 66238, Mexico; 3 Division of Cellular and Molecular Medicine, St George's Hospital Medical School, University of London, London SW17 0RE, UK

## Abstract

*Streptococcus pneumoniae* resides in the oxygen-rich environment of the upper respiratory tract, and therefore the ability to survive in the presence of oxygen is an important aspect of its *in vivo* survival. To investigate how *S. pneumoniae* adapts to oxygen, we determined the global gene expression profile of the micro-organism in aerobiosis and anaerobiosis. It was found that exposure to aerobiosis elevated the expression of 54 genes, while the expression of 15 genes was downregulated. Notably there were significant changes in putative genome plasticity and hypothetical genes. In addition, increased expression of *rgg*, a putative transcriptional regulator, was detected. To test the role of Rgg in the pneumococcal oxidative stress response, an isogenic mutant was constructed. It was found that the mutant was sensitive to oxygen and paraquat, but not to H_2_O_2_. In addition, the absence of Rgg strongly reduced the biofilm-forming ability of an unencapsulated pneumococcus. Virulence studies showed that the median survival time of mice infected intranasally with the *rgg* mutant was significantly longer than that of the wild-type-infected group, and the animals infected with the mutant developed septicaemia later than those infected intranasally with the wild-type.

## INTRODUCTION

*Streptococcus pneumoniae* is an aerotolerant Gram-positive bacterium that causes an array of diseases, including pneumonia, otitis media and meningitis (Kadioglu *et al.*, 2008). The ability to cause diseases in diverse *in vivo* environments suggests that the micro-organism is equipped with robust mechanisms to sense and adapt to changing environmental parameters, such as variation in the concentration of oxygen. Indeed, the importance of proteins involved in the oxidative stress response for pneumococcal biology has been demonstrated in various studies (Auzat *et al.*, 1999; Giuliodori *et al.*, 2007; McDougald *et al.*, 2002; Yesilkaya *et al.*, 2000).

Bacteria employ mainly enzymic mechanisms to eliminate the damaging effects of oxidative stress, such as superoxide dismutase (Auzat *et al.*, 1999; Giuliodori *et al.*, 2007; McDougald *et al.*, 2002; Yesilkaya *et al.*, 2000), NADH oxidase (Auzat *et al.*, 1999; Giuliodori *et al.*, 2007; McDougald *et al.*, 2002; Yesilkaya *et al.*, 2000), catalase (Rocha *et al.*, 1996), glutathione peroxidase, glutathione reductase (Vergauwen *et al.*, 2003), thiol peroxidase (Cha *et al.*, 2004) and alkyl hydroperoxidase (Paterson *et al.*, 2006). The presence of one or several of these enzymes has been shown in many pathogenic bacteria, and they have been linked to microbial virulence (Cha *et al.*, 2004; Cianciotto, 2001; Yesilkaya *et al.*, 2000).

The pneumococcus has to deal with changing concentrations of oxygen during infection. Although some aspects of the pneumococcal response to oxidative stress have been described, information generally is fragmentary. The presence of pneumococcal superoxide dismutase (Yesilkaya *et al.*, 2000), NADH oxidase (Auzat *et al.*, 1999) and alkyl hydroperoxidase (Paterson *et al.*, 2006) has been shown, and their importance in the pneumococcal oxidative stress response has been established (Auzat *et al.*, 1999; Spellerberg *et al.*, 1996). Previously, we have shown that *S. pneumoniae* synthesizes a manganese-dependent superoxide dismutase, SodA. A *sodA* pneumococcal mutant is more susceptible to the oxidative stress induced by paraquat and less virulent in a mouse model of pneumonia (Yesilkaya *et al.*, 2000). Additionally, some of the pneumococcal surface antigens have also been linked to oxidative stress response, such as pneumococcal surface antigen A, inactivation of which renders pneumococci susceptible to H_2_O_2_ (Tseng *et al.*, 2002; McCluskey *et al.*, 2004).

*In silico* analysis of the pneumococcal genome revealed other genes whose products have been implicated in oxidative defence in other bacteria. However, their role in the pneumococcal oxidative response remains to be investigated. These genes include *tpX* (thiol peroxidase), SP0313 (glutathione peroxidase), *nth* (endonuclease III), and genes encoding several heat-shock proteins, such as *groEL*, *groES* and *dnaK* (Tettelin *et al.*, 2002). Significantly though, the pneumococcus has neither catalase nor oxidative stress response regulators that are present in other micro-organisms, for instance *oxyR*, *soxRS* and *perR* (Tettelin *et al.*, 2002). Hence, the first aim of this work was to study the global oxidative stress response of *S. pneumoniae.*

Noteworthy within the pneumococcal oxidative stress response was *rgg* overexpression. This putative transcriptional regulator is present in several other Gram-positive bacteria, and has been shown to be involved in regulation of glutamate-dependent acid tolerance, the synthesis of glucosyltransferase, utilization of non-glucose carbohydrates, prophage induction and oxidative stress (Chaussee *et al.*, 2003, 2004; Kreikemeyer *et al.*, 2003; Pulliainen *et al.*, 2008). However, there is no report on the contribution of Rgg to pneumococcal biology. Hence the second aim of this work was to define a role for Rgg in the pneumococcus. For this, we constructed an isogenic mutant strain and tested its response to oxidative stress, biofilm formation and virulence.

## METHODS

### Bacterial strains and growth conditions.

*S. pneumoniae* type 2 strain D39 and its unencapsulated derivative R6 were used in this study. Routinely, *S. pneumoniae* strains were grown in brain heart infusion (BHI) broth or tryptic soy broth (TSB), or on blood agar plates supplemented with 5 % (v/v) defibrinated horse blood, at 37 °C. Sicard's defined medium (Sicard, 1964) was used for oxygen, paraquat and H_2_O_2_ susceptibility assays in order to eliminate the scavengers of reactive oxygen species (ROS) that may be found in rich medium. Where appropriate, spectinomycin (100 μg ml^−1^) was added to the culture medium.

For micro-aerobic growth, tightly closed culture tubes were used. Anaerobic growth conditions were created by using GasPak Anaerobic System Envelopes (BD BBL) in a jar containing a platinum catalyst, and anaerobiosis was monitored with an anaerobic indicator (BD BBL Dry Anaerobic Indicator Strips). With this method, at the time of inoculation, there was 10 % (v/v) dissolved oxygen, which decreased to 2 % (v/v) by 2 h and to 0 % by the time the bacteria reached mid-exponential phase at ∼4 h (OD_500_ ∼0.6).

For aerobic growth, culture flasks were placed in a water bath at 37 °C with a stream of filtered air bubbled through the culture. The oxygen concentration remained at 20 % (v/v) until mid-exponential phase (OD_500_ ∼0.6), then the concentration dropped to ∼10 % by stationary phase (OD_500_ ∼1.3).

### Biofilm formation assay.

The biofilm formation assay was done in flat-bottomed polystyrene tissue-culture plates (96-well plates; Sarstedt) as described previously (Oggioni *et al.*, 2006). Frozen pneumococcal cultures (about 1×10^8^ c.f.u. ml^−1^) were diluted 1 : 100 in 200 μl TSB and 100 ng ml^−1^ competence-stimulating peptide (Inbios). After incubation at 37 °C for 18–24 h, the supernatant was decanted and wells were washed four times with 200 μl ice-cold TSB to remove planktonic cells. Then, 100 μl TSB containing 10 % (v/v) glycerol was added and the biofilm was detached by 2 s sonication in a sonicating water bath. The biofilm-forming cells were quantified by plating on blood agar.

### H_2_O_2_ survival assay.

Mid-exponential phase cultures (50 μl) (OD_500_ ∼0.6), were centrifuged to remove supernatant and resuspended in an equal volume of PBS (pH 7.0). The bacteria were mixed with 50 μl H_2_O_2_ (Sigma) to give a final concentration of 20 or 40 mM H_2_O_2_, and the reaction was incubated at 37 °C for 5 or 20 min. The control reactions contained bacteria and PBS. c.f.u. were determined by serial dilution and plating on blood agar plates. The results were expressed as percentage survival relative to the control.

### Paraquat-susceptibility assay.

The pneumococcal inoculum was prepared as described above, and exposed to either 0.05 or 0.1 mM paraquat for 1 h. c.f.u. were determined by serial dilution and plating on blood agar plates. The results were expressed as percentage survival relative to the control.

### RNA extraction and purification.

The extraction of RNA was done as described previously (Stewart *et al.*, 2002). The pneumococcal cultures were grown in BHI broth under aerobic or anaerobic conditions until mid-exponential phase (OD_500_ ∼0.6), and were immediately mixed with GTC solution [5 M guanidine isothiocyanate, 0.5 % (w/v) sodium *N*-lauryl sarcosine, 25 mM trisodium citrate (pH 7.0), 100 mM 2-mercaptoethanol and 0.5 % (v/v) Tween 80]. The bacteria were harvested by centrifugation at 5000 ***g*** for 20 min and the pellet was resuspended in 1.2 ml RNAzol (Sigma). This was transferred to a RiboLyser blue matrix tube (Hybaid) and processed in a RiboLyser (Hybaid) at 6.5 power setting for 45 s. The RNA was extracted first with chloroform and then precipitated with 2-propanol. Finally, the RNA was treated with amplification grade DNase I (Invitrogen) before purification with an RNeasy Mini kit (Qiagen).

### Microarray experiments.

The R6 strain was used for microarray experiments. Microarray slides were obtained from the Bacterial Microarray Group at St. George's Hospital Medical School, University of London. The SPv1.1.0 array consisted of spotted PCR products that represent all of the genes in the *S. pneumoniae* TIGR4 and R6 genomes. The array design is available in  μG@Sbase (accession number A-BUGS-14; http://bugs.sgul.ac.uk/A-BUGS-14) and also ArrayExpress (accession number A-BUGS-14). The experimental procedures for microarray analysis followed previously reported methodology (Stewart *et al.*, 2002), as briefly described below.

RNA (2–10 μg) extracted from strain R6 was labelled with either Cy3 or Cy5 dCTP (Invitrogen) during cDNA synthesis using SuperScript II reverse transcriptase (Invitrogen) and random primers (Invitrogen). Cy3- and Cy5-labelled cDNAs were mixed and purified using a MiniElute Purification kit (Qiagen). Microarray slides were pre-hybridized with freshly prepared pre-warmed pre-hybridization solution of 3.5× SSC (3 M NaCl, 300 mM sodium citrate, pH 7.0) containing 0.1 % (v/v) SDS and 5 mg BSA ml^−1^ for 20 min at 65 °C. The slides were thoroughly rinsed, first in sterile nano-pure water for 1 min and then in 2-propanol for a further 1 min. The excess liquid on the slide was removed by centrifugation at 500 ***g*** for 5 min and hybridized within 1 h.

The hybridization mixture contained purified Cy3/Cy5-labelled cDNA samples in 4× SSC and 0.3 % (v/v) SDS in a total volume of 23 μl. This was heated to 95 °C for 2 min and left to cool slightly at room temperature. The slide was then incubated at 65 °C in the dark for 16–20 h in a hybridization cassette. After this, the slide was washed for 2 min three times, first with 1× SSC, 0.5 % (v/v) SDS, and then twice with 0.6× SSC. Finally, the excess liquid was removed by centrifugation at 500 ***g*** for 5 min.

### Analysis of microarrays.

The microarray slides were scanned using an Axon GenePix 4000A microarray scanner, which utilizes GenePix 5.1 software (Molecular Devices) for identification and for a visual analysis of the quality of the spots. Each spot was visually assessed as ‘good’, ‘bad’ or ‘not found’. ‘Good’ spots could be clearly distinguished from the background, and had an ‘average’ size. ‘Bad’ spots were affected by artefacts, such as lines of dye across the array, or were very small or too big, while ‘not found’ indicated the apparent absence of hybridization. The raw intensity data obtained from five independent experiments were normalized and further analysed using GeneSpring 7.3 software (Agilent Technologies). Data were subjected to LOWESS intensity-dependent normalization. This normalization method is commonly used for two-colour experiments and corrects for dye incorporation artefacts (GeneSpring user manual). Statistically significant changes in gene expression between aerobic and anaerobic samples were determined as *t*-test *P* values <0.05 after Benjamini and Hochberg false discovery rate correction (Green & Diggle, 2007). Genes of interest were further identified by requiring >twofold differences in all five samples analysed. In addition, the microarray results for selected genes whose expression significantly altered in aerobiosis relative to anaerobiosis, were verified and confirmed by real-time quantitative RT-PCR (qRT-PCR) in order to ensure that dye affinity did not bias the results.

### qRT-PCR.

Two independent RNA preparations were used for qRT-PCR analysis. First-strand cDNA synthesis was performed on approximately 1 μg DNase-treated total RNA, immediately after isolation, using 200 U SuperScript II reverse transcriptase (Invitrogen) and random hexamers at 42 °C for 55 min (Yesilkaya *et al.*, 2006). cDNA (15 ng) was amplified in a 20 μl reaction volume that contained 1× SYBR Green PCR master mix (Applied Biosystems) and 3 pmol of each primer (Table 1). The transcription level of specific genes was normalized to *gyrB* transcription, amplified in parallel with sp0806F and sp0806R primers. The reactions were performed in triplicate using the following cycling parameters with a MX4000 real-time PCR cycler (Stratagene): 1 cycle of 10 min at 95 °C followed by 40 cycles of 30 s at 95 °C, 1 min at 55 °C, and 30 s at 72 °C. The results were interpreted using the comparative cycle threshold (C_T_) method (Livak & Schmittgen, 2001).

### Construction of *rgg* mutants.

Two *rgg* mutant strains were constructed: an insertion mutant in the encapsulated D39 strain background, designated *rggM*, and an insertion–deletion mutant in the unencapsulated D39 derivative R6, designated Δ*rgg*. The mutations were made in two strain backgrounds in order to assess whether the presence of capsule would mask the effects of the *rgg* mutation on biofilm formation, and the encapsulated mutant was constructed to assess the effect of the mutation on virulence. The list of the primers that were used to construct the mutants is given in Table 1. To construct the *rggM* mutant, the chromosomal region between nucleotides 1 921 440 and 1 923 338, containing the *rgg* (SPD1952) coding sequence, was amplified with the RGGF and RGGR primers. The amplified products were incubated with *Himar1* transposase (Lampe *et al.*, 1996) and plasmid pR412, which contains the *mariner* mini-transposon conferring spectinomycin resistance (Martin *et al.*, 2000). Then, the *in vitro*-mutagenized DNA was transformed into the pneumococcus using competence-stimulating peptide (Alloing *et al.*, 1996). Transformants were selected for spectinomycin resistance, and insertion of the resistance cassette was confirmed by sequencing, and PCR by combining transposon-specific primers MP127 or MP128 with chromosomal primers RGGF or RGGR, respectively.

To construct the *rgg* insertion–deletion mutant in R6, the splicing by overlap extension (SOEing) PCR method was used (Horton *et al.*, 1990). The 487 bp fragment containing 31 nt of *rgg* and its upstream sequence was amplified with C-SOE-F and D-SOE-R, while a 561 bp fragment containing 8 nt of *rgg* and its downstream sequence was amplified using A-SOE-F and B-SOE-R primers. A 1184 bp spectinomycin-resistance gene was amplified from pDL278 with specSOE-F and specSOE-R, which incorporated ends complementary to B-SOE-R and C-SOE-F primers. Finally, equimolar amounts of amplicons containing DNA upstream and downstream of *rgg,* as well as the spectinomycin-resistance cassette, were mixed, and fused by PCR using A-SOE-F and D-SOE-R primers. The amplicons were gel-purified (Qiagen) and transformed into R6 as above, and the mutation was confirmed by PCR and sequencing.

### *In vivo* virulence studies.

Female MFI outbred mice, weighing 30 to 35 g, were obtained from Harlan Olac. A standardized inoculum was prepared as described previously (Yesilkaya *et al.*, 2000). Mice were lightly anaesthetized with 2.5 % (v/v) fluothane (Zeneca) over oxygen (1.5–2 l min^−1^). A 50 μl sample of PBS containing 5×10^5^ c.f.u. *S. pneumoniae* was administered into the nostrils. Mice were monitored for clinical signs (progressively starry coat, hunched appearance and lethargy) (Morton, 1985) for 7 days by the researchers and animal housing facility staff who were blinded to the identity of mice, and those that reached the severely lethargic stage were accepted to have reached the end point of the assay and were killed humanely. The time to this point was defined as ‘survival time’. Mice that were alive for 7 days after infection were deemed to have survived the infection. To express the disease signs numerically, a score of 2, 4 or 6 was given if the mouse was hunched, had a starry coat, or was lethargic, respectively. Median survival time was analysed by the Mann–Whitney *U* test.

To detect bacteria in the blood, approximately 20 μl venous blood was obtained from intranasally infected mice at predetermined time points after infection. Viable counts in blood were determined on selective and non-selective media.

## RESULTS

### Analysis of microarray data

The microarray data indicated that the expression of 69 genes was affected in aerobiosis compared with anaerobiosis: 54 genes were upregulated and 15 were downregulated (Table 2). The microarray results were also confirmed by qRT-PCR for selected genes (Table 2).

Expression of *sodA* (which encodes a manganese co-factored superoxide dismutase) and *tpx* (which encodes a thiol peroxidase) increased in aerobiosis. This is consistent with their known importance in the oxidative stress response of bacteria (Atack *et al.*, 2008; Yesilkaya *et al.*, 2000). Moreover, upregulation of the gene for the oxygen-sensitive pyruvate formate lyase (*pfl)* in anaerobiosis (Neves *et al.*, 2005) indicated that the anaerobic condition was adequately established in this study. The expression of *groES*, *groEL*, *dnaK*, *ahpD*, SP0313 (glutathione peroxidase) and *nox*, which are known to take part in the oxidative stress response in other bacteria, was unaltered in aerobiosis, presumably due to use of air in this study instead of H_2_O_2_ or paraquat (Chang *et al.*, 2005, 2006; Mostertz *et al.*, 2004). On the other hand, the transcription of a number of other genes not directly involved in the oxidative stress response displayed an altered pattern of expression. These were hypothetical and conserved hypothetical genes of unknown function and those annotated by homology as genes for genome plasticity, ATP binding cassette (ABC) transporters, ribonucleotide reductase system, Bgl and phosphotransferase system (PTS)-related genes (Lanie *et al.*, 2007; Tettelin *et al.*, 2002).

### Involvement of *rgg* in the oxidative stress response

The Rgg family of transcriptional regulators is found in Gram-positive bacteria (Chaussee *et al.*, 2003). These include the Rgg and RggD of *Streptococcus gordonii*, GadR of *Lactococcus lactis*, Rgg or RopB of *Streptococcus pyogenes* and MutR of *Streptococcus mutans* (Chaussee *et al.*, 2003; Vickerman *et al.*, 2003). In other streptococci, Rgg-type regulators have been linked to regulation of adherence, colonization, biofilm formation, carbohydrate and amino acid metabolism, as well as the oxidative stress response (Chaussee *et al.*, 2003, 2004; Pulliainen *et al.*, 2008; Vickerman *et al.*, 2003).

**Fig. 1. f1:**
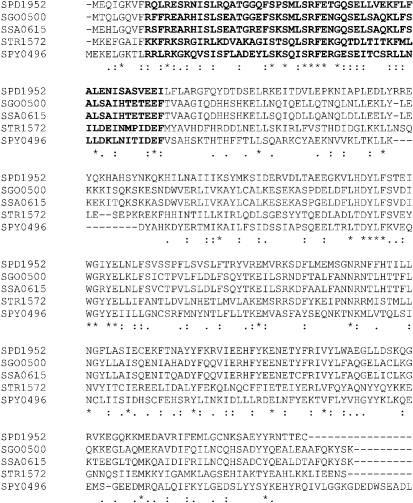
Alignment of deduced amino acid sequences of Rgg/MutR family proteins. SPD1952, *S. pneumoniae* D39 Rgg (accession number YP817340); SGO0500, *S. gordonii* RggD (accession number 5599480); SSA0615, *S. sanguinis* RggD (accession number 4806754); STR1572, *S. thermophilus* MutR (accession number 3166136); and SPY0496, *S. pyogenes* MutR (accession number 900726). Multiple alignments were performed using the clustal w program. Asterisks indicate that the aligned residues are identical. Substitutions assumed to be conservative or semi-conservative by clustal w are indicated by (:) and (.), respectively. The helix–turn–helix motif is indicated in bold type.

**Fig. 2. f2:**
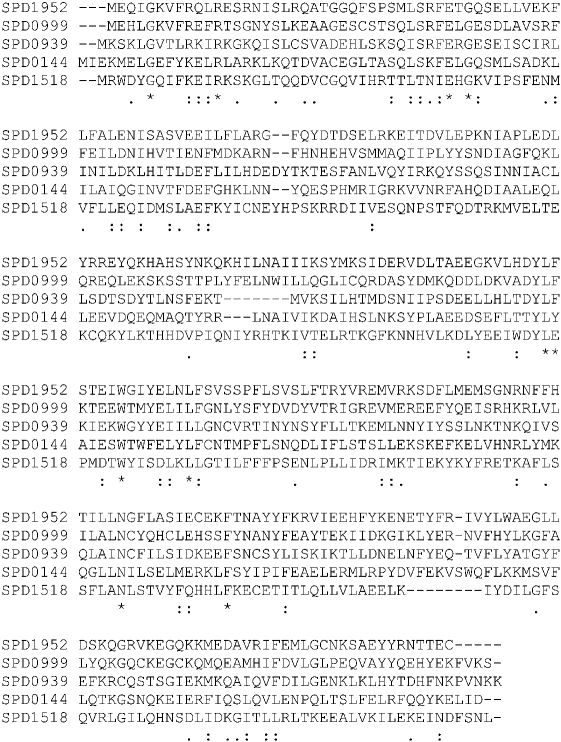
Alignment of deduced amino acid sequences of SPD1952 paralogues. Accession numbers: Q04MT5 (SPD0144), Q04KN2 (SPD0939), Q04KH4 (SPD0999), and Q04J65 (SPD1518). The alignment was performed and formatted as described in the legend to Fig. 1.

In aerobiosis, SPD1952 (SP2123 and SPR1933 in TIGR4 and R6 genomes, respectively) expression was elevated 11-fold compared with in anaerobiosis. This gene was designated *rgg*, since its deduced amino acid sequence shares significant identity with other Rgg-type transcriptional regulators in *S. gordonii* (46 %), *Streptococcus sanguinis* (45 %), *Streptococcus thermophilus* (32 %) and *S. pyogenes* (26 %) (Fig. 1). Characteristically, Rgg-type transcriptional regulators contain a helix–turn–helix motif in the N-terminal region (Dmitriev *et al.*, 2008). This motif in SPD1952 shares a sequence identity of 59, 57, 38 and 30 % with the same motif in *S. gordonii*, *S. sanguinis*, *S. thermophilus* and *S. pyogenes,* respectively (Fig. 1). *rgg* is present in all sequenced pneumococcal genomes (www.ncbi.nlm.nih.gov), but in TIGR4 SP2123 contains a frameshift in its sequence, possibly leading to an inactive protein. In addition to SPD1952, there are an additional four *rgg* paralogues, SPD0144, SPD0939, SPD0999 and SPD1518, which exhibit 24, 26, 33 and 23 % homology to SPD1952, respectively (Fig. 2) (Dmitriev *et al.*, 2008). Because of the importance of Rgg in streptococcal biology (Chaussee *et al.*, 2004, 2003; Kreikemeyer *et al.*, 2003; Pulliainen *et al.*, 2008), it was decided to study the role of the annotated *rgg* in the context of the pneumococcal oxidative stress response.

### The *rgg* mutant exhibits susceptibility to oxidative stress

As expression of *rgg* was found to be elevated in aerobiosis, we reasoned that in the absence of Rgg, pneumococci should be more susceptible to oxidative stress. The results showed that the mutant grew as well as the wild-type parental strain under anaerobic conditions in Sicard's defined medium (0.48±0.05 h^−1^, *n*=4). However, under aerobic conditions, the mutant demonstrated a significant growth impairment. While the growth rate of the parental D39 strain was 0.45 h^−1^ (±0.03, *n*=4), the growth rate of *rggM* was significantly lower: 0.34 h^−1^ (±0.03, *n*=4) (*P*<0.05). In terms of yield, there was no difference between *rggM* and D39 (data not shown).

**Fig. 3. f3:**
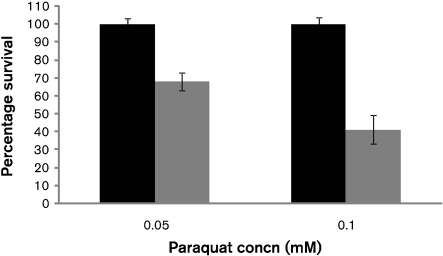
Effect of paraquat on the survival of pneumococcal strains. Black bars, D39; grey bars, *rggM*. Error bars indicate sd.

To determine whether Rgg is involved in protection against internally produced superoxide, the pneumococcal strains were exposed for 1 h to 0.05 or 0.1 mM paraquat, a redox-active compound that generates intracellular superoxide in the presence of oxygen. Neither concentration affected the survival of D39 compared with unexposed controls (Fig. 3) (*P*>0.05). However, the percentage survival of *rggM* declined to 68 % (±3, *n*=3) and 41 % (±4, *n*=3) after exposure to 0.05 and 0.1 mM paraquat, respectively (*P*<0.05).

**Fig. 4. f4:**
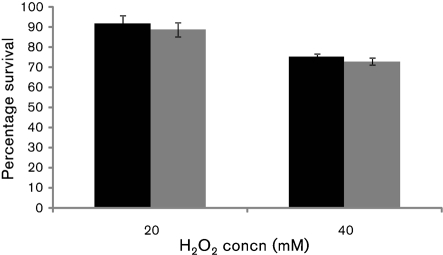
Susceptibility of pneumococcal strains to H_2_O_2_. Black bars, D39; grey bars, *rggM*. Error bars indicate sd.

The pneumococcus produces H_2_O_2_ under nutrient-rich and aerobic conditions, predominantly by the action of pyruvate oxidase (Pericone *et al.*, 2003). Although the amount of H_2_O_2_ produced by *S. pneumoniae* is lethal for many micro-organisms, pneumococcal growth is not inhibited at high concentrations of endogenously produced H_2_O_2_ (Pericone *et al.*, 2003). However, the factors affecting H_2_O_2_ resistance are not known entirely. Due to the involvement of Rgg in the oxidative stress response, we tested whether Rgg has any role in protection against H_2_O_2_. It was found that exposure to 20 and 40 mM H_2_O_2_ for 20 min resulted in a similar level of killing of both *rggM* and the parent D39 strain (*P*>0.05) (Fig. 4). For example, while 92 % (±4.2, *n*=3) of D39 and 89 % (±3.4, *n*=3) of *rggM* survived 20 mM H_2_O_2_, 40 mM exposure resulted in a decline in survival to 75 % (±1.1, *n*=3) and 73 % (±1.9, *n*=3) for *rggM* and wild-type, respectively. This suggested that Rgg does not afford protection against H_2_O_2_.

Analysis of expression of genes downstream of *rgg* indicated the absence of any polar effect in the mutant. Firstly, the microarray data did not show that the expression of downstream genes was affected by different oxygen concentrations, ruling out their involvement in the oxidative stress response and co-regulation with *rgg*. Secondly, qRT-PCR analysis confirmed that the expression of SPD1951, the gene immediately downstream of *rgg,* was not significantly different in *rggM* (1.4±0.2-fold, *n*=3) compared with D39, in which the normalized (with *gyrB*) expression was assumed to be 1 (*P*>0.05).

### *sodA* and *tpx* expression in *rggM*

As *sodA* and *tpx* expression was elevated in aerobiosis, the involvement of Rgg in the regulation of these genes was investigated. qRT-PCR analysis of *sodA* and *tpx* in *rggM* showed that while *sodA* expression was not greatly different (1.6±0.2-fold, *n*=3) from that in D39, *tpx* expression was significantly higher in *rggM* (2.6±0.2-fold, *n*=3) than in D39 (*P*<0.05), implying that *tpx* is under the negative control of Rgg. As thiol peroxidase is important for removal of H_2_O_2_, upregulation of *tpx* in *rggM* may partly explain why *rggM* survival was not affected by H_2_O_2_.

### Role of Rgg in biofilm formation

Biofilm-forming ability has an important link to pneumococcal virulence, as has recently been reported (Muñoz-Elías *et al.*, 2008). Moreover, the involvement of Rgg in attachment of other bacteria has been described (Samen *et al.*, 2006); hence, we decided to determine the biofilm-forming ability of *rggM*. Both encapsulated strains, D39 and *rggM*, formed biofilm equally well, although the number of biofilm-forming bacteria was 100 times less for both strains relative to R6 (data not shown), presumably due to the effect of capsule which can reduce biofilm formation (Moscoso *et al.*, 2006). However, when capsule was absent, an effect of loss of Rgg was apparent, since the proportion of biofilm-forming cells in Δ*rgg* (2.1±0.3) was nearly 50-fold less than that of the parental strain (100±24) (*P*<0.05).

### Virulence test

**Fig. 5. f5:**
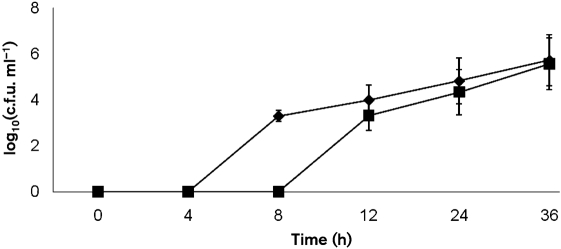
Numbers of pneumococci in the blood of mice infected intranasally with D39 (♦) or *rggM* (▪). Each point is the mean log_10_(c.f.u. ml^−1^) from 20 mice, except at 4 and 8 h, which are from 10 mice. Error bars indicate sem.

In a mouse model of pneumonia it was found that *rggM* was less virulent than the D39 parental strain. The median survival time of the *rggM*-infected cohort (65±29 h) was significantly longer than that of the wild-type-infected cohort (45±9 h) (*P*<0.001). Moreover, the onset of disease signs was faster in the wild-type-infected group [disease scores for 24, 36 and 48 h were 2.2 (±0.4), 4.4 (±0.5) and 5.1 (±0.4), respectively, *n*=20 for each datum point] compared with the *rggM*-infected cohort [disease scores for 24, 36 and 48 h were 0.7 (±0.3), 2.4 (±0.4) and 2.8 (±0.5), respectively, *n*=20 for each datum point] (*P*<0.05 at 24 and 36 h, and *P*<0.01 at 48 h). While bacteraemia occurred sometime between 4 and 8 h after intranasal infection in the wild-type-infected group, bacteraemia was at least 4 h later in the *rggM*-infected group (Fig. 5). However, once in the blood, there was no difference in the rate of increase in numbers of the two strains, suggesting that mutation of *rgg* leads to attenuation of the passage of the pneumococcus from the lungs to the blood.

## DISCUSSION

The pneumococcus is exposed to varying levels of oxygen in different host environments; hence, efficient strategies are required to adapt to changes in oxygen concentration. This is needed not only for averting the damaging effects of oxygen radicals but also because variation in oxygen concentration has direct effects on essential traits of pneumococci, such as the regulation of capsular polysaccharide synthesis (Weiser *et al.*, 2001), competence development (Echenique & Trombe, 2001) and membrane composition (Pesakhov *et al.*, 2007).

We determined the pneumococcal oxidative stress response by exposing the pathogen to a constant flow of environmental oxygen, rather than superoxide-generating agents or hydrogen peroxide. This ensured that an environment similar to that present in the nasopharynx was created, and the induction of a potential shock response was avoided (Mongkolsuk & Helmann, 2002). In this study, the total number of genes with altered expression was much smaller than in similar studies profiling oxidative stress responses of various other bacterial species (Chang *et al.*, 2005, 2006; Mostertz *et al.*, 2004). While 3.3 % of the pneumococcal genome-coding capacity exhibited altered expression, in other studies this has ranged from 4 to 9 %. For example, it was 4 % in *Pseudomonas aeruginosa* PAO1 (Chang *et al.*, 2005), 7 % in *Bacillus subtilis* 168 (Mostertz *et al.*, 2004), and 9.1 % in *Staphylococcus aureus* NCTC8325 (Chang *et al.*, 2006). This could be due to a number of reasons, including genuine interspecies differences in response to oxidative stress, the nature of the stimuli, the microarrays used, and the technical and analytical procedures that were employed.

The results showed that the pneumococcus responds to oxidative stress, firstly by attempting to neutralize ROS and then by reducing the burden of damage. Through the induced expression of genes for enzymes known to deplete ROS, such as superoxide dismutase and thiol peroxidase, the pneumococcus can avert the damaging effect of ROS, while repression of transport of iron into the cell (i*.*e*.* by downregulation of ABC transporters), might prevent the formation of hydroxyl radicals. In addition, the genes essential for the synthesis of genetic material (i.e. ribonucleotide reductase system genes), are downregulated, perhaps protecting the pneumococcal nucleic acids from the potential mutagenic effect of ROS. The genes involved in carbohydrate metabolism were also upregulated in aerobiosis, presumably to meet the increased demand for metabolic energy under stress.

In aerobiosis, the expression of several *bgl* genes was increased. These genes are involved in carbohydrate utilization, transport and regulation (Amster-Choder & Wright, 1997; Amster-Choder, 2005). In addition we observed overexpression of PTS genes, notably SP2036 and SP2038 (the same as SPR1847 and SPR1849, respectively). PTS systems have a role in attachment to surfaces in *S. mutans* and *Vibrio cholerae* (Abranches *et al.*, 2006; Houot & Watnick, 2008), but further work is required to establish whether these genes have a similar function in *S. pneumoniae*. Although upregulation of *bgl* and PTS system genes was observed in aerobiosis, differential expression may also be due to modification of metabolism for adaptation to aerobic growth conditions.

In bacteria, ABC transporters are mainly implicated in nutrient uptake and removal of toxins and antibiotics (Ulijasz *et al.*, 2004). In aerobiosis, the expression of several ABC transporter genes decreased, including *fatD*, *fatC*, *fatE* and *fatB*, which form an iron transport operon (Ulijasz *et al.*, 2004). This may be an adaptive response to oxidative stress by reducing levels of intracellular iron, consequently preventing the production of hydroxyl ion via the Fenton reaction (Touati, 2000).

The ribonucleotide reductase systems provide deoxyribonucleoside triphosphates needed for DNA synthesis. Their synthesis is influenced strongly by oxygen in other bacteria (Torrents *et al.*, 2001). In this study we found upregulation of three deoxyribonucleoside triphosphate synthesis genes, *nrd*, in anaerobiosis. *nrdG* has been found to be upregulated during infection of blood and the central nervous system, where the concentration of oxygen is low (Orihuela *et al.*, 2004), suggesting the importance of the ribonucleotide reductase system in pneumococcal anaerobic growth.

The proportion of hypothetical and conserved hypothetical genes with altered expression in aerobiosis (39 %) outweighed the genomic representation of these classes: 32.7 and 26 % of genome-coding capacity in type 2 and 4 strains, respectively (Lanie *et al.*, 2007; Tettelin *et al.*, 2002). This suggests that some of these genes have a direct role in the oxidative stress response. Indeed, increased expression of some of the hypothetical and conserved hypothetical genes has also been documented in various other experimental models (Martin-Galiano *et al.*, 2005; Orihuela *et al.*, 2004).

The second line of defence against oxidative stress may be mediated by competence and genome plasticity, as we found increased expression of several pneumococcal genes linked to competence and transposition upon exposure to air. Induction of genetic diversity is a way of bacterial adaptation, and can result in enhanced virulence. Oxygen is known to control competence development (Echenique *et al.*, 2000), and induction of competence increases pneumococcal pneumonia (Oggioni *et al.*, 2006). When the pneumococci were subject to aerobiosis, *comF* [also referred to as *comFC* (sp2207)], *cglD* (sp2050) and *cglC* (sp2051) were upregulated. *cglC* and *cglD* belong to the *cgl* locus, which has been described as crucial for the production of competence (Pestova & Morrison, 1998). On the other hand, *comF* (Rimini *et al.*, 2000) is believed to be important for the late stages of competence (Dagkessamanskaia *et al.*, 2004), as it is in *B. subtilis* (Ogura *et al.*, 2002).

The Rgg-mediated response was identified as the third mechanism against oxidative stress. We defined a role for pneumococcal *rgg* in oxidative stress, biofilm formation and virulence. It was found that mutation of *rgg* renders pneumococci more susceptible to oxidative stress. A similar phenotypic effect has also been reported for an *rgg* mutant of *S. pyogenes*, which is more sensitive to paraquat (Chaussee *et al.*, 2004). We found that *rgg* mutation did not alter pneumococcal susceptibility to peroxide; however, mutation of an *rgg*-like gene renders *S. pyogenes* resistant to peroxide (Pulliainen *et al.*, 2008), probably due to upregulation of thiol peroxidase in *rggM*, as we determined in this study.

The virulence studies demonstrated that mutation of *rgg* renders the pneumococcus less virulent than the wild-type strain, and sepsis occurs later in an *rggM*-infected cohort than in the wild-type-infected group. This could be due to the mutant's susceptibility to oxidative stress and consequently a decreased ability to colonize and invade. However, given that Rgg has a comprehensive effect on various metabolic pathways, such as amino acid and carbohydrate metabolism (Chaussee *et al.*, 2003, 2004; Pulliainen *et al.*, 2008), the reduction in virulence is unlikely to be wholly explained by susceptibility to oxidative stress. Hence, a comprehensive understanding of the role of Rgg in pneumococcal biology requires the study of various regulatory networks affected by this protein, including regulation of the oxidative stress response and genome plasticity. In contrast to the pneumococcus, an *rgg* mutant of *S. pyogenes* has been found to be more virulent than its parental strain in a murine intraperitoneal infection model (Chaussee *et al.*, 2003, 2004; Pulliainen *et al.*, 2008). The discrepancy between results is likely to be due to the divergent roles of Rgg in these micro-organisms. Although both the pneumococcal and the *S. pyogenes rgg* have a helix–turn–helix motif at the 5′ end of the coding sequence that is characteristic of Rgg transcriptional regulators (Dmitriev *et al.*, 2008), the sequence similarity between these two genes is low (18 %).

Overall, the results of this project indicated that the oxidative response in *S. pneumoniae* is not limited to overexpression of a few genes but it is manifested by the concerted action of complex and integrated pathways.

## Figures and Tables

**Table 1. t1:** Primers used in this study

**Primer**	**Primer sequence (5′–3′)**
**Mutation primers**	
A-SOE-F	AAGGATCCAAGAGGCATACGCAGGCCAC
B-SOE-R	GTATTCACGAACGAAAATCGATCACGACCGAATGTTGAATATCTGC
C-SOE-F	GCATAACTTTCTCGTCCATATCGGACTTTTCCAATCTGCTCCATAG
D-SOE-R	AAGGATCCAAGTGATTGGCCTGCTTAACGAG
MP127	CCGGGGACTTATCAGCCAACC
MP128	TACTAGCGACGCCATCTATGTG
RGGF	AAGAGGCATACGCAGGCCAC
RGGR	AAGTGATTGGCCTGCTTAACGAG
specSOE-F	GATCGATTTTCGTTCGTGAATAC
specSOE-R	CGATATGGACGAGAAAGTTATGC
**Primers for gene expression analysis**	
spr0186F	ACGGGGAAAGTCAGGACACTGG
spr0186R	TGGGTACTACCAGATGGCGTCC
spr0288F	ACCGCACTCCCTGCCAACGAG
spr0288R	ACTTTGACGACAACCAAAGCCGTTC
spr0674F	TGGCGGACACTTGAACCACG
spr0674R	ACGAGTTGTTGCTGCTGCAGTG
spd0674F	ACACCCTGAAATCGGTGAAG
spd0674R	AGCTGTTTTCTCGGGAGTCA
sp0806F	TCGTGTGGCTGCCAAGCGTG
sp0806R	GGCTGATCCACCAGCTGAGTC
spr1349F	ACCTGCTTTGAAGACACCTCATGT
spr1349R	TCAAGATAGCCCAAGCTTGCTCA
spr1367F	TGCTGAGGTGGTCAATCAGGCTCT
spr1367R	AGTTCCCCTTTCCCAAGAGGCA
spr1495F	AGTCGGCGACAAGGCGCTTG
spr1495R	CGACGTGTTTGAGTTGAGCAGATGC
spr1685F	TGCCTTGGTAGGACCTATGGCCT
spr1685R	ATCAAGGCTTGCCCCAAGGT
spr1932F	ATGCTTTTGGTGCTTCGATT
spr1932R	CTCGTGTAATCTGCCCGAAT
spr1933F	TCACTGAGACAAGCAACCGGAGG
spr1933R	ACCTCTTGCCAGAAAGAGGATCTCC
spr2012F	TCGTTCAGAGCGCTTGGGGACAG
spr2012R	ACATCCTTAGCACCAGCTTCTTCC

**Table 2. t2:** Genes identified by microarray that undergo a change of expression when *S. pneumoniae* R6 is grown under aerobiosis compared with growth anaerobically The genes are divided into groups according to their predicted products. Genes expressed more in anaerobiosis are indicated with an arrow (↓). The annotation was based on the R6 genome (indicated by an ‘SPR’ prefix) (Hoskins *et al.*, 2001), and genes specific for strain TIGR4 are indicated by an ‘SP’ prefix (Tettelin *et al.*, 2002).

**Category and gene ID**	**Gene**	**Description**	**Fold change**	***P* value**	qRT-PCR confirmation*
**Known oxidative stress-related**					
SPR0674	*sodA*	Superoxide dismutase	8	0.001	3.6 (0)
SPR1495	*tpx*	Thiol peroxidase	12	0.003	9.4 (0.2)
**Hypothetical and conserved hypothetical**					
SPR0084		Conserved hypothetical protein	4	0.001	
SPR0186		Hypothetical protein	↓12	0.002	0.3 (0.09)
SPR0289		Hypothetical protein	↓12	0.00007	
SPR0429		Conserved hypothetical protein	↓7	0.007	
SPR0643		Hypothetical protein	4	0.003	
SPR0965		Hypothetical protein	3	0.001	
SPR0966		Conserved hypothetical protein	4	0.0001	
SPR0967		Conserved hypothetical protein	4	0.0001	
SPR0968		Hypothetical protein	15	0.00001	
SPR0895		Conserved hypothetical protein	4	0.002	
SPR1129		Hypothetical protein	19	0.00005	
SP1332		Hypothetical protein	16	0.00008	
SP1346		Conserved hypothetical protein	4	0.00002	
SPR1280		Hypothetical protein	6	0.0000002	
SPR1313		Hypothetical protein	3	0.0001	
SPR1348		Hypothetical protein	4	0.006	
SPR1535		Conserved hypothetical protein	5	0.0007	
SPR1537		Hypothetical protein	2	0.001	
SPR1572		Hypothetical protein	3	0.0000003	
SPR1623		Hypothetical protein	3	0.003	
SP2004		Hypothetical protein	4	0.007	
SPR1830		Hypothetical protein	3	0.0001	
SPR1857		Hypothetical protein	5	0.0002	
SPR1858		Hypothetical protein	6	0.00008	
SPR1859		Hypothetical protein	4	0.0007	
SPR1914		Hypothetical protein	3	0.0008	
SPR1983		Conserved hypothetical protein	6	0.001	
**Bgl and PTS-related**					
SPR1843		Transcriptional regulator, BglG family	9	0.002	
SPR1845	*sga*	Hexulose-6-phosphate isomerase	5	0.0008	
SPR1846	*sgh*	Hexulose-6-phosphate synthase	6	0.0002	
SPR1847	*PTS-EII*	PTS system, IIA component	6	0.01	
SPR1849	*PTS-EII*	PTS system, membrane component	6	0.0004	
**ABC transporters**					
SPR1281	*ABC-NBD*	ABC transporter, multidrug efflux	4	0.001	
SPR1289	*ABC-N/P*	ABC transporter, ATP-binding	13	0.007	
SPR1290	*ABC-N/P*	ABC transporter, ATP-binding	4	0.00006	
SPR1293	*ABC-NBD*	ABC transporter, ATP-binding	14	0.00009	
SPR1294		ABC transporter	5	0.0006	
SPR1684	*fatD*	Iron-compound ABC transporter	↓6	0.003	
SPR1685	*fatC*	Iron-compound ABC transporter	↓14	0.002	0.12 (0.01)
SPR1686	*fatE*	Iron-compound ABC transporter	↓6	0.002	
SPR1687	*fatB*	Iron-compound ABC transporter	↓10	0.002	
SPR1735		ABC transporter permease protein	6	0.00003	
**Ribonucleotide reductase system**					
SPR0183	*nrdD*	Anaerobic ribonucleoside triphosphate reductase	↓4	0.000002	
SPR0185	*nrdG*	Anaerobic ribonucleoside triphosphate reductase activating enzyme	↓7	0.00001	
SPR1065	*nrdE*	Ribonucleoside-diphosphate reductase	↓3	0.000001	
**Competence**					
SPR1861	*cglD*	Competence protein CglD	6	0.000003	
SPR1862	*cglC*	Competence protein CglC	4	0.0003	
SPR2012	*comFC*	Competence protein ComF	12	0.007	0.4 (0.09)
**Transposons and IS elements**					
SPR0644		Degenerate transposase	3	0.0005	
SP1314		IS*66* family element, Orf1	4	0.003	
SPR1349		IS*630*-Spn1 related, Orf2	3	0.02	2.2 (0.01)
SPR1367		Transposase	3	0.00005	2.1 (0.1)
SPR1985		Degenerate transposase	6	0.007	
SPR2016		Degenerate transposase	10	0.0001	
SPR0957		Tn*5252*, relaxase, truncation	2	0.01	
**Others**					
SPR0184		Acetyltransferase, GCN5-related *N*-acetyltransferase (GNAT) family	↓7	0.00002	
SPR0290	*gno*	Oxidoreductase	↓14	0.0005	
SPR0288	*kdgK*	Carbohydrate kinase, PfkB family	↓15	0.002	0.2 (0.13)
SPR0307	*clpL*	ATP-dependent Clp protease	↓8	0.0001	
SPR0415	*pfl*	Formate acetyltransferase	↓4	0.004	
SPR0630	*thiE*	Thiamin-phosphate pyrophosphorylase	4	0.0002	
SPR0960	*mutR*	Similar to positive transcriptional regulator MutR	3	0.0009	
SPR0918	*asd*	Aspartate beta-semialdehyde dehydrogenase	5	0.0001	
SPR1239	*amy*	Alpha-amylase precursor	3	0.001	
SP1759		Preprotein translocase	6	0.003	
SP1767		Glycosyltransferase	12	0.03	
SPR1933	*rgg*	Positive transcriptional regulator of glucosyltransferase	11	0.0003	3.2 (0.03)

*The microarray results were verified by measuring the expression of selected genes by qRT-PCR. The sd is indicated in parentheses.

## Abbreviations

- ABC, ATP binding cassette
- PTS, phosphotransferase system
- qRT-PCR, quantitative RT-PCR
- ROS, reactive oxygen species
